# Supplementary material for: Are carbon emissions trading and green financial instruments synergistic? -Comprehensive quantitative research based on content analysis
Source: PLoS One. 2024 Mar 7;19(3):e0298601. doi: 10.1371/journal.pone.0298601 (PMC10919601; doi:10.1371/journal.pone.0298601)
Supplement: S1 Data — (ZIP) [file pone.0298601.s001.zip › Data/地级市Z_F工作报告.zip!/╣π╢1⁄2/╤⌠╜¡╩╨/└┤╫╘╣1⁄2╓┌║┼í╛┬φ┐╦╩2╛▌═°í┐_2017.docx]

各位代表：

现在，我代表市人民政府，向大会报告政府工作，请予审议，并请政协各位委员和其他列席人员提出意见。

一、2016年及过去五年工作回顾

过去一年，面对经济下行压力，市政府认真贯彻落实中央、省和市委的决策部署，以推进供给侧结构性改革为主线，突出“三大抓手”，统筹推进稳增长、促改革、调结构、惠民生、防风险各项工作，经济社会保持平稳发展。预计全市地区生产总值1342亿元，增长7%；人均地区生产总值53268元，增长6.2%；规模以上工业增加值490亿元，增长7%；农业总产值380亿元，增长3.5%；社会消费品零售总额632亿元，增长8.2%；城镇居民人均可支配收入25097元，增长8.7%，农村居民人均可支配收入13697元，增长9.2%。一年来，重点做了以下工作：

（一）推动经济稳定增长。加大有效投资，推进重大基础设施、重大产业项目和重大民生工程建设，完成固定资产投资636亿元，其中能源项目投资128亿元。扩大消费需求，电子商务交易总额89亿元，增长35%。开展对外贸易，建成阳江（汇达）跨境电商快件分拣清关中心，支持木森等企业开拓国外市场，发展跨境电商等外贸新业态，进出口总额130亿元。

（二）推进产业转型升级。发展先进制造业、高新技术产业，战略性新兴产业产值191.1亿元，占规模以上工业总产值比重10%，提高4.5个百分点。加快发展现代服务业。海陵岛被评为中国“十大美丽海岛”。全市接待游客突破1800万人次，旅游总收入突破200亿元。积极发展现代农业。新增南药、花卉等特色经济作物5.07万亩，春砂仁成为全省首批被立法保护的中药材。新增国家级农民合作社示范社2家、市级农业龙头企业11家和现代农业园区6个。海洋渔业稳步发展，渔业总产值160.7亿元，增长3.8%。获批全国首家五金刀剪知识产权快速维权中心，新增国家级高新技术企业6家、省级科技企业孵化器2个，创建首个省级众创空间。

（三）抓好重点项目建设。建立市领导挂点联系督办重点项目责任制，现场协调解决用地、用海、资金等问题。加强与政策性银行合作，新增政府购买服务融资57亿元。83个市重点项目完成投资273亿元，完成年度计划投资111.5%。阳江核电站3号机组、广青合金热送设备技改等10个项目建成投产，海陵岛大桥等项目扎实推进，闸坡现代渔港等项目动工建设。

（四）打好创卫攻坚会战。以创建国家卫生城市为抓手，推进中心城区扩容提质，滨海新区“六路一桥”、漠阳湖公园等项目加快建设。实施一批市政项目，振兴路一期、城南西路、南山公园等建设进展顺利，“三廉公园”、龙山公园建成开放，城市功能进一步完善。加温鼓劲，攻坚克难，市领导挂钩社区，成立八大专责小组，市直区直单位派专人包干社区，实行“门前三包”责任制，推进创卫“十大工程”，创卫基本通过国家暗访，市容市貌更加整洁靓丽。

（五）全面对接珠海帮扶。加强产业园区合作共建，累计投入11亿元完善园区基础配套设施，新建和改扩建道路17.6公里。全年引进产业项目58个，总投资163亿元；新投产项目29个。加强海港、空港对接，阳江合山机场改扩建一期完成，阳江港货物吞吐量突破2000万吨。推进社会事业领域合作，百家学校医院对接成效明显。珠海对口帮扶阳江在省第一轮评估考核中获优秀等次。

（六）保障改善人民生活。省、市十件民生实事全面完成。城镇新增就业4.19万人，城镇职工五大险种参保人数143万人次。国家义务教育发展基本均衡县实现全覆盖，新增学位4440个。市妇幼保健院新院一期基本建成，11间镇卫生院标准化建设扎实推进。阳江加入海上丝绸之路保护与申遗中国城市联盟。建设基层公共文化体育综合服务中心22个。推进精准扶贫，2.55万贫困人口实现脱贫。完成“六五”普法工作，一村（社区）一法律顾问全覆盖。加强综治信访维稳工作，强化安全生产监管，社会治安持续向好，社会大局总体稳定。

（七）加强生态环境保护。实施清洁生产技术改造企业24家，完成4万千瓦电机节能改造任务，新增新能源公交车100辆。淘汰黄标车及老旧车5614辆。建成城北污水处理厂。关闭禁养区养殖场53家，治理禁建区养殖场30家。完成造林10.65万亩，建设碳汇林1.63万亩、生态景观林带129.3公里，新增湿地公园3个。

（八）着眼打基础利长远。超前谋划重要交通、重点园区、城市提质和支柱产业等重大项目，及早启动高新区扩园修编、滨海新区向南向海路网谋划、“两江四岸”环境改造等重点项目前期工作，加强项目储备，建立动态项目库，增强工作的前瞻性和主动性。利用土地中期评估成果，推动“三规合一”，统筹经济社会发展。

各位代表！刚刚过去的2016年，是本届政府收官之年。五年来，全市上下团结一心，努力拼搏，推动经济社会发展取得显著成就。

——这五年，综合实力迈上新台阶。地区生产总值、人均地区生产总值、一般公共预算收入、固定资产投资、社会消费品零售总额年均分别增长10.8%、10%、11.2%、10.9%和11.1%，分别是2011年的1.7倍、1.7倍、1.67倍、1.6倍和1.7倍，2013年人均地区生产总值在粤东西北地区率先超过全国同期平均水平。

——这五年，产业发展取得新成效。三次产业比例由20.942.836.3调整为17.542.739.8。五金刀剪产业成为首个年产值超500亿元产业集群，国家新能源基地、高端不锈钢产业基地初具规模。海陵岛大角湾海上丝路旅游区被评为全省首个滨海类国家5A级景区，百利广场、新都汇、君怡广场等大型商业综合体建成开业。建成现代农业园区50个，水产品总产量位居全省前列。

——这五年，基础设施实现新突破。推进“一港两桥三路”等重要交通基础设施建设，公路总里程突破1万公里。完成110千伏及以上输变电工程21项、农村电网改造项目2310个。加固达标海堤115公里，除险加固水库102宗，改造中小灌区25宗，重建漠阳江双捷引水工程拦河闸。建成高标准基本农田92.63万亩。移动宽带普及率为62.1%，光纤入户率达55.6%。

——这五年，城乡面貌得到新提升。滨海新区加快发展，完成阳东撤县设区，中心城区面积从40.51平方公里扩大到68.07平方公里，城镇化率从46.97%提高到50.5%。新建和改造市政道路227条，完成金山植物公园建设、鸳鸯湖改造等景观工程，建成区绿地率达40.07%，成功创建国家园林城市。县城和中心镇加快建设，农村人居环境不断改善，创建美丽乡村220个，整治自然村1497个，村庄垃圾收集点实现全覆盖。城市环境空气质量位居全省前列，漠阳江水环境质量总体达国家Ⅱ类标准，连续5年获省环保责任考核优良以上等次。

——这五年，改革开放增添新活力。市县镇三级权责清单全面公布，非行政许可事项全部取消，市级累计取消下放行政许可事项307项。建成省网上办事大厅阳江分厅、6个县（市、区）分厅以及47个镇（街道）、825个村（居）的网上办事平台。组建市人民医院医院集团，在全省率先完成市县镇三级卫生计生服务机构整合。“珠中江+阳江”等区域合作深入开展。

——这五年，民生事业达到新水平。民生支出占一般公共预算支出的比重超过60%。城镇登记失业率控制在2.6%以内。成功创建省教育强市，省教育强镇覆盖率达100%。新建一批社区休闲体育公园，新增省级以上非物质文化遗产项目6个。建成公租房4236套，改造农村低收入住房困难户住房26237户。完成两轮扶贫开发“双到”任务。连续4届荣获“全国双拥模范城”称号。成为首批国家防震减灾示范城市。工青妇、关工委、社科、残联、慈善、老龄事业持续发展，民族宗教、统计、审计、外事侨务、打私、人防、供销、档案等工作取得新成效。

五年来，我们始终加强政府自身建设。落实法治政府建设实施纲要，依法行政考核连续3年被省评为良好。认真执行市人大及其常委会决议决定，密切与市政协沟通协商，自觉接受法律监督、民主监督和社会监督，办理人大代表议案和建议298件，办理政协建议案和提案872件，办复率100%。中央八项规定精神有效落实，全市党政机关和参公单位会议费及“三公”经费财政拨款总支出累计下降50%以上。认真落实党风廉政建设责任制，惩治和预防腐败体系建设扎实推进，查处了一批违纪违法案件。

各位代表！过去五年成绩来之不易，这是市委正确领导的结果，是市人大、市政协大力支持和有效监督的结果，是全市人民团结拼搏的结果。在此，我代表市人民政府，向全市广大干部群众，向各民主党派、工商联、无党派人士，向驻阳江部队、武警官兵、中央和省驻阳江单位，向离退休老同志，向关心支持阳江发展的社会各界人士和朋友们，表示崇高的敬意和衷心的感谢！

在肯定成绩的同时，我们也清醒地看到存在的困难和问题，主要有：经济总量不大，主导产业不突出，产业层次不高，创新能力不强；经济下行压力较大，部分企业生产经营困难，2016年一般公共预算收入、固定资产投资等经济指标出现负增长；城乡环境建设有待提升，城市管理不够精细，农村脏乱现象较为普遍；人才规模总量偏小，高层次人才缺乏；改善民生任务依然繁重，全面建成小康社会仍有短板；依法行政水平有待提高，个别地区和部门的干部存在不敢担当、慢作为现象。对这些困难和问题，我们将采取有力措施，认真加以解决。

二、今后五年奋斗目标和主要任务

今后五年，是我市全面建成小康社会的决胜阶段。虽然当前经济下行压力较大，但综观国内外形势，世界经济仍处于缓慢复苏过程，我国经济发展回稳向好的基本面没有变；我省正深入实施粤东西北振兴发展战略，加大力度推进跨区域产业共建，着力推进珠三角与粤东西北一体化发展；我市经过建市近30年的发展积累，区位交通和产业体系不断完善，资源条件和后发优势不断凸显。特别是市第七次党代会，吹响了“以海兴市、绿色发展，为决胜全面小康、建设富美阳江而努力奋斗”的号角。只要我们坚定信心、保持定力，励精图治、勇往直前，就一定能够抓住机遇加快振兴发展，就一定能够补齐短板全面建成小康社会！

今后五年全市经济社会发展的总体要求是：全面贯彻党的十八大和十八届三中、四中、五中、六中全会精神,深入贯彻习近平总书记系列重要讲话精神，坚持稳中求进工作总基调，牢固树立和贯彻落实新发展理念，按照省委、省政府和市第七次党代会部署要求，以推进供给侧结构性改革为主线，以加快发展为第一要务，突出以海兴市、绿色发展，大力实施“六大举措”，全面建成小康社会，谱写富美阳江新篇章。

今后五年全市经济社会发展主要预期目标是：到2018年，力争与全省同步全面建成小康社会；到2021年，地区生产总值突破2000亿元，规模以上工业总产值突破3200亿元，一般公共预算收入突破90亿元，全体居民人均可支配收入达到2.8万元，人民幸福指数、文化发展活力、生态环境质量、社会治理能力明显提升。重点抓好六大任务：

（一）突出以海兴市，建设产业新城

以临港工业为主攻方向，重点提升五大产业集群，打造广东沿海临港工业新高地，力争到2021年，不锈钢、电力能源、装备制造、食品加工等产业发展成为年产值超500亿元产业集群。加快上马热轧、冷轧、铬合金原材料项目，引入下游深加工企业，延伸产业链，提高附加值，努力建成全国最大的不锈钢产业基地之一。加快阳江核电站、华厦阳西电厂、阳江抽水蓄能电站和太阳能光伏等能源项目建设，全面启动海上风电场项目，力争到2021年，全市能源项目总装机容量接近2000万千瓦，成为全省最大的能源基地。依托三峡新能源、中山明阳风电等龙头企业，以资源换产业，引入风机及配套生产企业，建设广东风电产业基地。促进喜之郎、厨邦食品等龙头企业增资扩产，做优豆豉、春砂仁制品等传统食品企业，加强食品产业招商，打造广东绿色食品生产基地。扶持和推动五金刀剪企业资源整合，提升工艺，开发新品，培育品牌，提升竞争力，拓展市场份额，做强中国五金刀剪基地。加快提升建材、家具、服装等传统产业，积极培育海洋生物医药、海洋工程装备、船舶制造等新兴产业。促进产业园区提质增效，把园区打造成为产业发展的主战场。围绕建设国内著名滨海旅游目的地，发挥海陵岛龙头带动作用，高起点谋划开发海陵岛东岛、阳东东湖星岛、阳西沙扒湾、阳春鸡笼顶、鹅凰嶂及南鹏列岛等旅游资源，着力引进大型旅游综合项目，开发高端旅游市场，建设国家蓝色旅游示范基地。力争到2021年，全市接待游客达3000万人次，旅游总收入超400亿元。构建区域性商贸物流中心，依托大海陵湾和阳江港，争取设立阳江港综合保税区，建设专业批发市场，大力发展航运和商贸流通业。以打造广东农海产品生产加工基地为抓手，大力发展现代农业和现代海洋渔业，加快发展农海产品精深加工，打响“中国南海渔都”品牌。

（二）突出扩容提质，建设滨海新城

加快滨海新区发展，抓好城南中心商务区、蓝色海岸、滨海生态公园、入海口绿廊生态景观带和沿海一级景观路等项目建设，推动中心城区向南向海，力争通过五年的努力，城市建设到海边，生活休闲在海边，建成名副其实的海滨城市。建设国际金融中心、市民中心，规划建设标志性建筑，打造城市新地标，彰显城市现代风貌。充分发挥山水自然生态优势，推进漠阳江、那龙河“两江四岸”滨水空间整理和景观提升，规划建设市区儿童公园和一批湿地公园、郊野公园、社区休闲体育公园，营造推窗见绿、碧水环绕的休闲城市。创新城市管理方式，推行城市网格化、精细化管理，使城市更加整洁有序。推进中心城区“三横四纵”城市快速交通体系建设，加快公共设施合理布局，促进江城与阳东、新城与旧城实现融合对接。强化阳春市区、阳西县城的城市副中心功能，重点提升城市综合承载力，建成各具特色的中小城市。统筹城乡一体化建设，大力开展农村人居环境综合整治，改善镇村生产生活环境。

（三）突出开放创新，建设活力新城

着力发展大交通。推进阳江港大开发，全力加快吉树作业区和全面启动丰头作业区建设，完善集疏运体系，加快建设深茂铁路阳江段、沈海高速公路阳江段“四改八”扩建、开平至阳春高速公路、海陵岛大桥、阳江港大桥等重大交通项目；积极谋划阳江支线运输机场、阳西军民两用港、海陵岛城市轻轨项目，积极争取广州至湛江客运铁路专线、张家界至三亚旅游高铁途经阳江、珠三角城际轨道西延至阳江，争取云浮（郁南）至阳江（阳西）高速公路、中阳高速西延至玉林（省界）高速公路早日立项，加快形成外联内畅的立体综合交通体系。以阳江高新区创建国家级高新区为总抓手，加快培育新型研发机构、创新中心和科技企业孵化器，积极创建一批工程中心、技术中心和重点实验室，大力引进高端人才、创新人才，加快发展高新技术产业。力争到2021年，全社会研发投入占地区生产总值比重明显提升，高新技术产业逐步发展成为主导产业，科技创新支撑引领作用显著增强。发挥阳江地处珠三角与大西南的重要交通节点，具有承东启西、“左右逢源”的地缘优势，积极参与大珠三角经济区、“珠中江+阳江”等区域合作，主动融入“一带一路”建设，实现加快崛起。进一步理顺市与市辖区、经济功能区的权责关系，完善各功能区的管理体制，增强发展新活力。

（四）突出海丝特色，建设文化新城

围绕创建全国文明城市目标，推进群众性精神文明创建活动，践行社会主义核心价值观，提升城市文明程度和市民素质。擦亮“南海I号”品牌，积极参与“海上丝绸之路·中国史迹”申报世界文化遗产，做好“海上丝绸之路”文化遗产的发掘保护，建设海上丝绸之路主题文化景区和海丝文化博物馆，提升阳江“海丝文化”影响力。着力挖掘保护漆器、风筝等非物质文化遗产，弘扬书画、音乐等地方传统特色文化，加强南恩路等历史文化街区和雅韶十八座等古村落的保护开发，谋划建设阳江文化名人纪念馆，彰显阳江文化特色。加强文化基础设施建设，推进公共文化服务标准化，打造城市“十分钟文化圈”和农村“十里文化圈”。设立文化产业发展基金，加快发展文化产业，促进文化与其他产业融合发展。打造海陵岛国际马拉松赛、帆船帆板赛、沙滩体育活动、风筝文化节、阳江“蚝美”美食节等特色活动品牌，提高阳江的知名度和美誉度。

（五）突出绿色发展，建设生态新城

以创建国家环保模范城市和国家森林城市为统领，推进生态文明建设，既要金山银山，又要绿水青山。抓好自然保护区建设，构建北部山区生态屏障。加强饮用水源保护工作，谋划大河水库供水项目。以《漠阳江流域保护条例》立法为契机，全面启动漠阳江流域综合治理，实现漠阳江水质明显提升。推进工业园区走绿色发展之路，开展重点行业、重点企业能效提升专项行动。完善环保基础设施，力争到2021年，全市新建污水处理厂16个，配套管网254.5公里，新增污水处理能力11.6万吨/日。实施雨污分流，力争三年内完成市区地下排污管网改造，实现城市污水收集和处理全覆盖，区域内黑臭水体得到根本性治理。加强“海绵城市”建设，到2021年20%以上建成区达到“海绵城市”建设目标要求。加强海域海岸使用管理，强化入海口和沙滩保护，抓好海洋生态示范区和美丽海湾建设。积极打造森林城市，推进森林围城、树木进城，营造绿树成荫、鸟语花香的美好家园。

（六）突出和谐共享，建设幸福新城

加大民生领域公共财政投入，推进基本公共服务均等化，加快补齐民生社会事业短板，力争到2021年，全市民生支出占一般公共预算支出的比重超过70%。做好就业和社会保障工作，五年城镇新增就业17.5万人。实施全民参保计划，全面落实城乡低保、农村五保政策，做到应保尽保。推进教育现代化先进市建设。积极发展学前教育，大力发展义务教育，协调发展高中阶段教育，提升发展高等教育，大力培养和引进学科带头人、骨干教师，提供更多的公办优质学位，让每个孩子能够上好学、读好书，力争到2021年，规范化幼儿园覆盖率90%，适龄儿童学前三年入园率达到95%以上，高中阶段教育毛入学率达到95%以上，新改扩建学校67所，新增学位6万个，建成一所本科高等院校。加快卫生强市建设。探索实施“医联体”新型医疗模式，完善市县镇村四级医疗卫生服务体系，加强镇卫生院、村卫生室标准化和信息化建设，加大资深专家、医学领军人物、急需紧缺型人才的引进力度，力争到2021年，全市有6所医院达到三级甲等标准，每个县(市、区）都有1所二级甲等以上标准医院，努力实现小病不出镇、大病不出县。加快人口健康信息化建设，力争到2021年，全市医疗卫生机构全部实现信息互联互通。实施精准扶贫、精准脱贫工程。到2018年，现行标准下贫困人口全部脱贫、贫困村全部出列。推进依法治市，加强和创新社会治理，为人民群众营造安居乐业的良好社会环境。

三、2017年工作安排

2017年，是新一届政府的开局之年，做好今年的工作意义重大。充分考虑各方面因素，今年经济社会发展的主要预期目标是：地区生产总值增长7%-7.5%，一般公共预算收入增长7%，规模以上工业增加值增长9%以上，固定资产投资增长10%，社会消费品零售总额增长8.5%，居民人均可支配收入增长高于经济增长，城镇登记失业率控制在3.5%以内，完成节能减排约束性指标任务。重点抓好以下八方面工作：

（一）促进经济较快发展

扩大有效投资。全年安排重点建设项目89个，总投资2277亿元，年度投资226亿元。强化市领导挂点联系督办重点项目责任制落实，加大服务协调力度，力促维达纸业首期等16个项目建成投产，加快三威汽配三期等48个续建项目建设，推进美味鲜食品生产项目一期等25个项目动工建设。启动第二批政府购买服务融资项目和PPP项目，举办“建设项目招商推介会”，推出一批基础设施项目，加快项目建设步伐。

拉动市场消费。推动大型商业综合体特色发展，加快阳江国际汽车商贸城、粤西农海产品批发市场等项目建设。举办旅游推介会，办好阳江“蚝美”美食节等特色节庆活动，多渠道扩大消费。推动刀博会创新发展，举办第16届刀博会，建设刀博会等线上线下交易平台，积极开展阳货网上行、阳货全国行等活动，扩大商品网上交易。发挥市农产品电商物流园的龙头带动作用，年内建成1个电子商务产业园、400个农村淘宝服务站点，力争打造1个全国电子商务进农村综合示范县、1个省级农村电子商务综合示范基地，打通农产品进城、工业品下乡双向通道。

促进对外贸易。支持企业开拓国际市场，扩大产品的出口份额。加强餐厨用品国家级示范基地和水产品省级示范基地建设，引导外贸企业加快转型升级，扶持培育出口品牌。积极发展外贸新业态，以阳江（汇达）跨境电商快件分拣清关中心业务为依托，加快本土跨境电商企业发展。优化进口结构，促进先进技术设备、关键零部件以及资源性原料进口。推进“三个一”等通关改革，深化“三互”通关合作，提高贸易便利化水平。

振兴民营经济。举办市长与民营企业家见面会，畅通政企沟通渠道。推进中小微企业公共服务平台建设，继续开展企业集中服务月活动，帮助企业解决实际困难和问题。着力解决企业融资难题，支持民营企业上市融资，对上市（挂牌）成功企业给予一次性奖励。发挥政策性融资担保公司和中小微企业信贷风险补偿金作用，为企业贷款提供担保。实施大企业大集团培育和“专精特新”中小企业发展计划，成立产业扶持基金，推动本地龙头企业发展，新增年主营业务收入超10亿元企业2家、超50亿元企业2家。

（二）发展壮大实体经济

推进产业园区发展。抓好珠海（阳江）产业园建设，港口片区新增征地3000亩，建成港口污水处理厂一期，启动二期工程，抓好疏港大道南、锦绣路等道路建设。启动建设港口片区生活配套区，完善公共服务设施。加强阳东、阳西和阳春产业园道路、供水供电、排水排污等基础设施建设，完善学校、医院等生活设施，提高园区承载力和吸引力。加大招商选资力度，力争全市4个省级产业园区年内承接珠三角产业转移项目55个以上，投资总额100亿元以上，园区规模以上工业增加值达到260亿元、增长20%以上。

推进主导产业发展。推动广青不锈钢深加工（热轧）和铬合金原材料配套生产线工艺技改项目第二季度动工，加快宏旺冷轧等不锈钢深加工项目签约。在佛山举办不锈钢制品专业招商会，引进一批深加工项目。加快发展电力能源产业，推动阳江核电站4号机组上半年投入商业运行，力促阳江抽水蓄能电站下库坝、中电建阳东宝山陆上风电场、高新区LNG综合利用等项目开工建设。大力发展先进装备制造业，推动三峡风电风机、粤水电装备项目动工，促进阳春新兴铸管一期达产，发展壮大风电装备、精密铸件和基础件等装备制造业。促进五金刀剪、食品加工等传统优势产业提质增效，落实技改事后奖补政策，引导65家规模以上工业企业实施技术改造，鼓励企业开展自动化和智能化生产线改造，提升企业竞争力和经济效益。

推进现代服务业发展。抓好海陵岛国家全域旅游示范区建设，创建阳西省级全域旅游示范区。完善海陵岛旅游配套设施，建成恒大儿童水上乐园，促进保利一线海悦酒店、玉露天街二期商业街加快建设，动工建设恒大国际会议中心，推动敏捷福朋喜来登酒店项目前期工作。加快东平、沙扒、春湾等旅游小镇建设。积极发展金融业，力争新增1家企业在主板或中小板上市、4家企业在“新三板”“新四板”挂牌。拓展普惠金融“村村通”试点工作，实现农村金融服务全覆盖。加快发展现代物流业，抓好三七物流、诚通物流及一批码头物流项目建设。统筹发展现代商贸业，力促东汇城、盈信广场等城市综合体建成开业。落实“互联网+”行动计划，推动互联网与服务业融合发展。

推进市属国企发展。做大做强市属国有企业，指导市恒财城投公司拓展融资租赁、商业保理、政策性担保等非银行金融业务，谋划部分板块股权改革，为上市创造条件，发展成为市属国有企业的龙头标杆企业。推动市水务集团统筹市域自来水安全供给，加快城乡供水一体化进程。支持市交投集团参与市政设施、交通项目和公益项目建设，筹措交通项目资本金。推动市漠阳置业集团加快农贸市场升级改造及下属企业发展混合所有制经济。

（三）推进城市向南向海

加强中心城区建设。完成金平路一期、城南片区“六路一桥”等主干道和漠阳湖公园一期工程，抓好深茂铁路阳江站综合交通枢纽工程建设，动工建设新阳路、郦阳路等市政道路和城南路、新阳河等沿路沿岸景观带，启动中心城区“三横四纵”城市快速路网、漠阳湖公园二期、滨海生态公园等前期工作。推进国际金融中心、中国移动大楼建设。抓好城南片区“海绵城市”试点项目。加快建设阳东“八路四贯通”路网，完善市政配套设施。投入22亿元，实施34个市政项目。完善市区路网主骨架，贯通江城与阳东、旧城区与滨海新区道路网络。推进旧城区、城中村等改造，完善旧城区市政基础设施，增加停车场和绿地空间。针对市区私人住宅较多的特点，探索建设“开放式街区”，缓解交通拥堵，增强社区活力。谋划建设漠地洞、罗琴山等郊野公园，启动市区文化公园、共青湖公园、阳东竹篙山森林公园规划建设和北湖公园升级改造，建设10个社区休闲体育公园。

加强基础设施建设。年度投资66亿元，推进铁路、高速公路、城市快速路、港口、通用机场建设。力争深茂铁路阳江段明年初建成。加快汕湛高速公路阳春段、海陵岛大桥、开平至阳春高速公路建设。力争阳江合山机场二期7月份全面动工。争取先行试点建设沿海一级景观路，抓好新325国道、省道277线改扩建，推动沈海高速公路开阳段扩建和服务区改造全面动工，启动阳江港大桥、10万吨航道疏浚和吉树、丰头作业区深水码头建设。投入12亿元推进一批电网工程建设，完善电力设施。加强信息基础设施建设，提高WIFI覆盖率。推动水利工程建设，完成8宗万亩以上海堤达标加固工程、2宗大型水闸重建工程、120公里中小河流二期治理，推动9宗中型水闸建设。

加强城市精细管理。以社区、单位为基础，推行城市网格化管理，制定奖惩措施，加强检查考核。全面落实“门前三包”责任制。抓好创卫工作，对照国家暗访发现的问题，落实整改措施，健全城市管理长效机制，力争创建成为国家卫生城市。推进“智慧城市”“数字城管”建设，运用电子网络、常态化巡查等手段，持续整治“六乱”现象，加大对电动车等车辆违章行为治理力度，提高城市精细化、智能化管理水平。

（四）做好农业农村工作

发展现代农业。抓好农业供给侧结构性改革。推动海洋渔业转型升级，新建50艘钢质渔船，建设50口深水网箱，扶持发展2个休闲渔业示范点，谋划推进“南海渔都”国际水产城项目。推进闸坡现代渔港高桩码头和重力式码头建设，加快溪头现代渔港（二级）项目建设进度，规划建设对岸渔港。发展现代农业园区，推进阳东国家、省级农业示范区建设，力争创建5个现代农业园区。完成10.48万亩高标准基本农田建设。

打造美丽乡村。以全面铺开“农村清洁工程”为抓手，着力改善农村人居环境，实现农村生活垃圾保洁覆盖率90%，完成30%自然村人居环境综合整治任务。在全市范围内整县推进农村村庄建设规划编制，提高自然村规划编制覆盖率。出台《阳江市村庄住宅建设管理意见》，推广《新农村住宅设计图集》，引导农民依规建设。建成阳东区省级新农村示范片，加快阳西、海陵省级新农村示范片建设。落实中心镇“九个一”工程，完成100个美丽乡村创建任务。

促进农民增收。引导农民以土地经营权、劳动、资金等要素入股参与产业化经营。积极发展新型经营主体，新增5家市级以上农业龙头企业。积极发展农产品加工业，大力发展休闲农业、生态农业、观光农业，创建2个省级休闲农业与乡村旅游示范点。加强农村、渔村劳动力技能培训，提高就业能力，增加农民、渔民工资性收入。

加快脱贫攻坚。计划投入2.9亿元专项资金，统筹推进贫困村“五个提升”，完成3.4万贫困人口脱贫任务。推动最低生活保障、残疾人保障和教育、医疗、住房等政策落地。加大产业帮扶力度，推进贫困户增收帮扶项目建设。积极开展小额扶贫信贷，着力解决贫困户贷款难问题。

（五）落实创新驱动战略

在培育高企上下功夫。启动高新技术企业培育计划，安排配套扶持资金，扶持有潜力的企业进入后备库，推动昌龙科技等12家企业入库培育。落实高新技术企业认定资助政策和税收优惠政策，引导入库企业积极对标、补齐短板，力争新增金恒达化妆工具有限公司等10家国家高新技术企业。

在建设平台上下功夫。支持广青金属科技有限公司工程技术研究中心创建国家级工程技术研究中心。推动阳江五金刀剪产业技术研究院、阳江职业技术学院、广青金属科技有限公司创建省级重点实验室。支持广东厨邦食品有限公司等年产值5亿元以上的企业设立研发机构，力促广东信海建筑有限公司建设省级新型研发机构。推动市农产品物流园科技企业孵化器创建省级科技企业孵化器。培育2家省级众创空间。

在引进人才上下功夫。完善和落实人才引进优惠政策，利用院士工作站等科研平台，加大柔性引才力度，力争1-2个创新团队入选省“扬帆计划”。推动重点企业与高校、科研院（所）建立人才合作关系，引进急需紧缺的高层次人才。深入实施人才培养工程，加快建设一支规模大、素质高、结构优的人才队伍。

（六）加大改革开放力度

深化供给侧结构性改革。妥善做好全市155家关停企业移交省股权托管中心及后续工作，扶持8家特困国有企业脱困。加快建立购租并举的住房制度，争取纳入省专业化住房租赁平台，对公租房建设、棚户区改造、农民回迁安置房等探索通过“团购”、发放“购房券”等方式进行安排。促进银行机构采取有效措施处置不良资产和降低不良贷款率，积极防范和稳妥处置金融风险。降低企业制度性交易成本、社会保险费、用电成本等，切实为企业减轻负担。加大财政投入，调动民间投资积极性，计划投入超100亿元推进补短板项目建设。

深化重点领域改革。以“一门式一网式”政府服务模式改革为抓手，纵深推进“放管服”改革，能下放的审批权限全部下放。抓好行政审批标准化建设，最大限度减少自由裁量权，优化办事流程，提高办事效率。深化商事制度改革，推进“多证合一”，加强事中事后监管、“双随机、一公开”监管。深化企业投资管理体制改革，落实《企业投资项目核准和备案管理条例》，分类实行核准管理或备案管理。抓好盐业体制、国有林场和供销社改革，推进农村土地承包经营权确权登记颁证工作。

深化区域交流合作。抓住第二轮对口帮扶机遇，加大产业共建力度，力争珠海（阳江）产业园年内不少于20个较大的工业项目落地建设，各县（市、区）加大力度引进亿元以上产业共建项目。加快融入“珠中江+阳江”经济圈，推进交通、产业、民生等方面深度合作。加强与湛江、茂名的对接，共同推进湛茂阳沿海经济带、北部湾经济圈建设，密切与海丝沿线国家和地区经贸往来。

（七）全面加强环境保护

抓好漠阳江流域综合治理。建立“河长制”，加强水环境治理。强化漠阳江流域面源污染防治，完成上游禁养区、禁建区养殖场整治，对不符合环保标准的企业实行搬迁或淘汰，严厉查处偷排偷放、超标排放行为。加快完善漠阳江流域沿线环保设施，提高污水处理能力。推进麻演灌渠等黑臭河段治理。抓好水源涵养林建设，提升漠阳江两岸绿化美化水平。

抓好污染防治。组建专职环保警察队伍，加大环保执法力度，严厉查处环境违法行为。完成饮用水水源保护区标志设置和隔离防护。加快市区城南污水处理厂二期和三江岛、长洲岛等污水处理厂建设。完成阳春省级污水处理示范县建设。完善镇级污水处理厂、生态型污水处理设施管网配套。推进村级污水处理设施建设，尽快提高农村污水处理率。抓好工业污染源、机动车尾气和工地、港口码头扬尘治理，基本完成“黄标车”淘汰任务，确保空气质量持续向好。摸清土壤污染底数，实施分级分类管理。以落实中央环保督查反映案件整改处置工作为抓手，解决一批环保突出问题。

抓好节能减排。严把节能评估和环保准入审查关，限制高耗能行业过快增长。抓好节能示范点建设，支持重点用能企业开展节能技术改造。以高新区、中山火炬（阳西）产业园为试点，推动产业园区开展循环化改造。实施清洁生产，力争完成清洁生产技改企业25家。完善市能管中心平台系统建设，尽快实现省、市、企业三级互联互通。

抓好生态建设。推进新一轮绿化阳江大行动，建设生态景观林带81.8公里、森林碳汇1.1万亩，新增森林公园9个，绿化美化乡村82个。抓好海洋生态系统保护修复，加快建设海陵岛、月亮湾国家级海洋公园，争取获批国家级海洋生态文明建设示范区。加强近岸海水质量监测监控，确保各项指标符合标准。推进矿山地质环境治理恢复。坚持依法和集约节约用地，保护好耕地。

（八）持续保障改善民生

抓好就业社会保障。促进高校毕业生等重点群体就业，城镇新增就业3.5万人，就业困难人员再就业2000人。抓好社会保险扩面征缴工作，进一步提高社保覆盖率。完善大病保险制度，提高城乡居民医保财政补助标准，稳步提升社会保障待遇水平。大力发展老龄事业，推进阳春市、阳西县养老院建设，鼓励社会力量参与发展各类养老机构。关爱农村留守儿童。完成28万人村村通自来水工程建设任务。新建保障性住房391套，完成农村危房改造10246户。

提升教育卫生水平。加快市县教育现代化创建工作，建设一批住宅小区配套小学、幼儿园和村级幼儿园，推进华南师范大学附属阳春实验学校、广东外语外贸大学附属阳江外国语学校和特殊学校标准化建设，推动阳江职业技术学院专升本。深入实施“强师工程”，提升教师队伍素质。开展生源地信用助学贷款，实现办理区域和办理对象全覆盖。深化医药卫生体制改革，重点推广市人民医院医院集团管理模式，加快实施分级诊疗制度。促进县镇医疗卫生机构一体化管理。抓好市人民医院新住院大楼、市中医院二期工程、市第三人民医院搬迁等项目，完成镇级卫生院标准化建设。积极探索社区卫生服务模式，开展社区卫生服务中心规范化建设，继续推进“家庭医生式服务”试点工作，让居民享受更好的医疗健康服务。加强计划生育管理服务，稳妥实施“全面两孩”政策。

增强城市文化实力。完成“南海I号”申遗保护展示工程任务。启动阳江文化名人纪念馆、市体育综合训练馆和城市规划馆筹建工作，建设民间工艺体验场所，改造市博物馆、市风筝馆，完善市美术馆等设施。筹划建设市文化创意产业园。办好南海开渔节、风筝文化节、美食节，举办国际马拉松赛、帆船帆板赛、市第六届运动会，扶持开展民俗表演等群众文化活动，丰富人民群众精神文化生活。

推进社会治理创新。推进平安阳江建设，深化信访制度改革，抓好社会矛盾化解，重点解决涉农、涉环保、涉房地产等领域社会矛盾问题。完成村（居）两委换届，落实城乡社区协商制度。完善社会治安打防控体系，加强反恐处突队伍建设，打击“两抢一盗”犯罪和电信网络诈骗犯罪。启动“七五”普法工作，加强公共法律服务体系建设。深入开展安全生产大检查，加强重点行业专项治理，强化消防监管，坚决遏制重特大安全事故发生。推进智慧食药监建设，加强食品药品事中事后监管。完善应急体系，提高社会公众应对自然灾害处置能力。全面推进全省气象现代化试点市建设。同时，抓好双拥共建、工青妇、社科、慈善、民族宗教、统计、外事侨务、打私、人防、供销、档案等工作。

补齐民生事业短板。把今年作为全面建成小康社会的攻坚之年，对照国家全面建成小康社会统计监测指标体系，主动作为，攻坚克难，补齐短板。投入34亿元办好提升底线民生保障、改善农村群众生产生活条件等十件民生实事，集中解决一批群众最关心、最直接、最现实的利益问题。

四、切实加强政府自身建设

（一）依法履行政府职能

落实阳江市法治政府建设实施纲要，坚持法定职责必须为，法无授权不可为。加强政府及部门法制机构建设，提高行政首长出庭应诉率。健全依法科学决策机制，发挥政府法律顾问和社会智库辅助决策作用。推进阳光政务建设。推动执法重心下移和执法事项属地化管理，切实做到严格规范公正文明执法。自觉接受人大、政协和社会各界的监督，认真办好人大代表议案、建议和政协建议案、提案。

（二）深入推进廉政建设

贯彻落实全面从严治党要求，履行好“一岗双责”，把纪律和规矩挺在前面。突出工程建设、土地出让、政府采购、财政资金使用、国有资产管理等重点领域和重要环节监管，加强预防腐败信息系统建设，推进廉政风险防控全覆盖。抓好反腐倡廉教育，强化源头治腐，加大违纪违法案件查办力度，严肃查处发生在群众身边的腐败问题，促进干部清正、政府清廉、政治清明。

（三）持续改进政务作风

巩固拓展党的群众路线教育实践活动、“三严三实”专题教育、“两学一做”学习教育成果，完善作风建设长效机制。加强各级行政服务大厅便民服务建设，完善公共服务事项目录和办事指南，加快推进部门间信息共享和业务协同，坚决砍掉各类无谓的证明和繁琐的手续。健全正向激励长效机制，建设敢于担当的干部队伍。严抓一些不作为、慢作为、乱作为的典型案例，实行问责，从严治理。

各位代表！潮平岸阔催人进，风正扬帆正当时。让我们紧密团结在以习近平同志为核心的党中央周围，在市委的坚强领导下，紧紧依靠和带领全市人民，振奋精神、只争朝夕，苦干实干、奋勇争先，为决胜全面小康、建设富美阳江而努力奋斗，以优异成绩迎接党的十九大胜利召开！
